# Supplementary material for: Role and mechanism of NCAPD3 in promoting malignant behaviors in gastric cancer
Source: Front Pharmacol. 2024 Apr 22;15:1341039. doi: 10.3389/fphar.2024.1341039 (PMC11070777; doi:10.3389/fphar.2024.1341039)
Supplement: Supplementary file 11 [file DataSheet2.ZIP › GSEA/immunologic/my_analysis.Gsea.1599462003817/GSE43955_1H_VS_20H_ACT_CD4_TCELL_WITH_TGFB_IL6_DN.html]

Details for gene set GSE43955\_1H\_VS\_20H\_ACT\_CD4\_TCELL\_WITH\_TGFB\_IL6\_DN[GSEA]

|  || Dataset | filtered\_dataset.sample\_info.cls#WT\_versus\_NCAPD3\_MUT |
| Phenotype | sample\_info.cls#WT\_versus\_NCAPD3\_MUT |
| Upregulated in class | NCAPD3\_MUT |
| GeneSet | GSE43955\_1H\_VS\_20H\_ACT\_CD4\_TCELL\_WITH\_TGFB\_IL6\_DN |
| Enrichment Score (ES) | -0.545743 |
| Normalized Enrichment Score (NES) | -2.6418128 |
| Nominal p-value | 0.0 |
| FDR q-value | 0.0040452625 |
| FWER p-Value | 0.018 |
Table: GSEA Results Summary

  

Fig 1: Enrichment plot: GSE43955\_1H\_VS\_20H\_ACT\_CD4\_TCELL\_WITH\_TGFB\_IL6\_DN      
 Profile of the Running ES Score & Positions of GeneSet Members on the Rank Ordered List

  

| SYMBOL | TITLE | RANK IN GENE LIST | RANK METRIC SCORE | RUNNING ES | CORE ENRICHMENT || 1 | 323 | APBB2 | 35 | 0.977 | 0.0440 | No |
| 2 | 55527 | FEM1A | 543 | 0.449 | -0.2894 | No |
| 3 | 203068 | TUBB | 736 | 0.358 | -0.4024 | No |
| 4 | 2317 | FLNB | 936 | -0.360 | -0.5202 | Yes |
| 5 | 4973 | OLR1 | 944 | -0.366 | -0.4994 | Yes |
| 6 | 3665 | IRF7 | 956 | -0.375 | -0.4808 | Yes |
| 7 | 9180 | OSMR | 981 | -0.394 | -0.4701 | Yes |
| 8 | 966 | CD59 | 1040 | -0.436 | -0.4810 | Yes |
| 9 | 3669 | ISG20 | 1097 | -0.475 | -0.4877 | Yes |
| 10 | 639 | PRDM1 | 1159 | -0.523 | -0.4946 | Yes |
| 11 | 26471 | NUPR1 | 1195 | -0.570 | -0.4794 | Yes |
| 12 | 4651 | MYO10 | 1213 | -0.588 | -0.4500 | Yes |
| 13 | 330 | BIRC3 | 1224 | -0.600 | -0.4147 | Yes |
| 14 | 2043 | EPHA4 | 1228 | -0.602 | -0.3742 | Yes |
| 15 | 837 | CASP4 | 1245 | -0.623 | -0.3416 | Yes |
| 16 | 1893 | ECM1 | 1250 | -0.637 | -0.2994 | Yes |
| 17 | 22822 | PHLDA1 | 1255 | -0.643 | -0.2567 | Yes |
| 18 | 7414 | VCL | 1308 | -0.712 | -0.2437 | Yes |
| 19 | 1649 | DDIT3 | 1341 | -0.779 | -0.2115 | Yes |
| 20 | 4233 | MET | 1358 | -0.821 | -0.1649 | Yes |
| 21 | 4734 | NEDD4 | 1369 | -0.856 | -0.1115 | Yes |
| 22 | 22936 | ELL2 | 1370 | -0.882 | -0.0490 | Yes |
| 23 | 5055 | SERPINB2 | 1397 | -1.088 | 0.0094 | Yes |
Table: GSEA details [plain text format]

  

Fig 2: GSE43955\_1H\_VS\_20H\_ACT\_CD4\_TCELL\_WITH\_TGFB\_IL6\_DN      
 Blue-Pink O' Gram in the Space of the Analyzed GeneSet

  

Fig 3: GSE43955\_1H\_VS\_20H\_ACT\_CD4\_TCELL\_WITH\_TGFB\_IL6\_DN: Random ES distribution      
 Gene set null distribution of ES for **GSE43955\_1H\_VS\_20H\_ACT\_CD4\_TCELL\_WITH\_TGFB\_IL6\_DN**
